# Dispersal in the sub-Antarctic: king penguins show remarkably little population genetic differentiation across their range

**DOI:** 10.1186/s12862-016-0784-z

**Published:** 2016-10-13

**Authors:** Gemma V. Clucas, Jane L. Younger, Damian Kao, Alex D. Rogers, Jonathan Handley, Gary D. Miller, Pierre Jouventin, Paul Nolan, Karim Gharbi, Karen J. Miller, Tom Hart

**Affiliations:** 1 Department of Zoology, University of Oxford, South Parks Road, Oxford, OX1 3PS UK; 2 Ocean & Earth Sciences, University of Southampton Waterfront Campus, European Way, Southampton, SO14 3ZH UK; 3 Department of Biology, Loyola University Chicago, 1032 W. Sheridan Road, Chicago, IL 60660 USA; 4 DST/NRF Centre of Excellence, Percy Fitzpatrick Institute of African Ornithology, Department of Zoology, Nelson Mandela Metropolitan University, South Campus, Port Elizabeth, 6031 South Africa; 5 Microbiology and Immunology, PALM, University of Western Australia, Crawley, WA 6009 Australia; 6 Centre National de la Recherche Scientifique, Centre d’Ecologie Fonctionnelle et Evolutive, UMR 5175 du CNRS, 1919 route de Mende, F-34293 Montpellier Cedex 5, France; 7 Department of Biology, The Citadel, 171 Moultrie St, Charleston, SC 29409 USA; 8 Edinburgh Genomics, Ashworth Laboratories, University of Edinburgh, Edinburgh, EH9 3JT UK; 9 Australian Institute of Marine Science, The UWA Oceans Institute, 35 Stirling Highway, Crawley, WA 6009 Australia

**Keywords:** Southern Ocean, Seabirds, Molecular ecology, *Aptenodytes patagonicus*, Dispersal, Genetic homogeneity, RAD-Seq, Colonisation, Gene flow

## Abstract

**Background:**

Seabirds are important components of marine ecosystems, both as predators and as indicators of ecological change, being conspicuous and sensitive to changes in prey abundance. To determine whether fluctuations in population sizes are localised or indicative of large-scale ecosystem change, we must first understand population structure and dispersal. King penguins are long-lived seabirds that occupy a niche across the sub-Antarctic zone close to the Polar Front. Colonies have very different histories of exploitation, population recovery, and expansion.

**Results:**

We investigated the genetic population structure and patterns of colonisation of king penguins across their current range using a dataset of 5154 unlinked, high-coverage single nucleotide polymorphisms generated via restriction site associated DNA sequencing (RADSeq). Despite breeding at a small number of discrete, geographically separate sites, we find only very slight genetic differentiation among colonies separated by thousands of kilometers of open-ocean, suggesting migration among islands and archipelagos may be common. Our results show that the South Georgia population is slightly differentiated from all other colonies and suggest that the recently founded Falkland Island colony is likely to have been established by migrants from the distant Crozet Islands rather than nearby colonies on South Georgia, possibly as a result of density-dependent processes.

**Conclusions:**

The observed subtle differentiation among king penguin colonies must be considered in future conservation planning and monitoring of the species, and demographic models that attempt to forecast extinction risk in response to large-scale climate change must take into account migration. It is possible that migration could buffer king penguins against some of the impacts of climate change where colonies appear panmictic, although it is unlikely to protect them completely given the widespread physical changes projected for their Southern Ocean foraging grounds. Overall, large-scale population genetic studies of marine predators across the Southern Ocean are revealing more interconnection and migration than previously supposed.

**Electronic supplementary material:**

The online version of this article (doi:10.1186/s12862-016-0784-z) contains supplementary material, which is available to authorized users.

## Background

Understanding the patterns and mechanisms of population structure is essential for successful species conservation [1]. For example, species with a high degree of population differentiation and limited dispersal among colonies may have a reduced ability to respond to unfavorable local environmental conditions [2] and may lose a large portion of their total genetic variation if local populations are lost or reduced [3]. Accurate data regarding the geographic boundaries of breeding populations and the degree of genetic exchange among them are therefore essential for species risk assessments and conservation planning, including to mitigate the effects of climate change. However, the extent of differentiation among natural populations of seabirds is difficult to predict and has been shown to vary widely among taxa [3, 4]. In general, seabirds are philopatric, with adults returning to natal sites to breed [5], and this behavior can be an isolating mechanism that acts as a barrier to gene flow. Seabirds that have large foraging range﻿s, or that breed at high latitudes, such as the polar regions, are thought to be the least likely to have differentiated populations as a result of recent range expansions and retained ancestral variation [3].

**Fig. 1:**
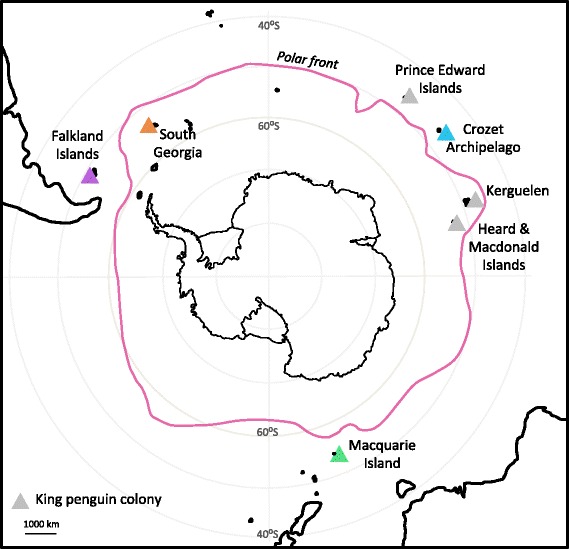
King penguin colony locations. *Triangles* indicate known king penguin colonies, with coloured triangles indicating the four colonies sampled for this study

King penguins (*Aptenodytes patagonicus*) are thought to be vulnerable to climate change impacts in the future [6, 7] and an understanding of their population structure is required to accurately model these impacts and make inferences about observed changes in population size. King penguins congregate in large breeding colonies on coastal ice-free ground on sub-Antarctic islands between 45 ° and 55 ° south [8] (Fig. 1). Numbers have been increasing across their range over the past several decades [8–11], following historic anthropogenic exploitation during the late 19th to early 20th centuries when they were slaughtered en masse for the blubber oil industry [12]. The global population of king penguins is now conservatively estimated at 1.6 million breeding pairs and still increasing [8].

Owing to their large and growing population size across most of their range, king penguins are currently listed as being of Least Concern on the IUCN’s Red List of Threatened Species [13] although there have been concerns that harvest may have resulted in a population bottleneck that would have reduced genetic variation and hence their adaptive capacity. Furthermore, king penguins will face new challenges in the coming decades as climate change alters their marine foraging habitat. The most immediate threat posed by climate change to king penguin populations is the southward shift of the Polar Front and deepening of the thermocline; both secondary to warming of the Southern Ocean’s surface waters [7]. King penguins forage almost exclusively at the Polar Front during the summer breeding season [14–16], as a result of the combination of predictably high prey abundance and ideal diving conditions that they find at the front [17, 18]. As sea surface temperatures increase with climate change, the position of the Polar Front is shifting to the south, and this is predicted to double the king penguin’s travelling distance to their preferred foraging grounds by 2100 [19]. The coincident deepening of the thermocline means the penguins must also dive deeper to reach their prey [7]. A study at the Crozet Islands has already demonstrated the impact that warming waters can have on king penguin numbers, with a population decline of 34 % associated with an anomalously warm year in 1997 [7]. In light of the potential threats to king penguin populations, accurate data regarding their population structure are needed [8]. Specifically, to monitor population sizes in relation to environmental impacts we must first understand what constitutes a genetic breeding population of king penguins.

There have been no studies of genetic population structure of king penguins across their breeding range to date. A decade-long study at one colony in the Crozet Islands found that 77 % of juvenile king penguins returned to their natal colony [20]. This suggests that the species is largely philopatric, however, even low numbers of dispersing individuals could be sufficient to homogenise populations [21]. King penguins possess a remarkable mobility, regularly conducting round-trips in excess of 3200 km from breeding colonies to forage in Antarctic waters during the winter months [15]. However, the average distance between the pairs of breeding sites in our study is 6500 km and the colonies are distributed longitudinally, whereas most of the king penguin’s foraging movement is latitudinal [16, 22]. This suggests that frequent dispersal among breeding sites should be unlikely. In spite of this, incidences of long-distance dispersal have been documented, with birds tagged on the Crozet Islands resighted resting or molting at Marion Island (900 km away) [23], Kerguelen Island (1500 km away), Macquarie Island (5600 km away) [24] and Heard Island (1740 km away) [25]. It should also be noted that any genetic differentiation that arose during the founding of colonies would be expected to persist for a very long time (i.e. thousands of generations) in a species with such a large effective population size and rapid population growth rate [26]. Previous studies of population structure in other penguin species revealed a remarkable lack of differentiation across thousands of kilometers, including in emperor penguins (*Aptenodytes forsteri*) [27, 28] and Adélie penguins (*Pygoscelis adeliae*) [29, 30]. This is in contrast to gentoo penguins (*Pygoscelis papua*) [31] and chinstrap penguins (*Pygoscelis antarctica*) [32], which demonstrated moderate to low genetic differentiation across similar distances. Both emperor and Adélie penguins have almost continuous circumpolar distributions [33, 34] that may faciliate migration, whereas king penguin colonies are scattered distantly across the sub-Antarctic (Fig. 1).

Overall, king penguins are a highly mobile marine species with huge potential for dispersal; however, genetic divergence among colonies may exist as a result of nonphysical barriers, such as philopatry, local adaptation or isolation by colonisation [35]. We therefore hypothesised that breeding colonies on different archipelagos would constitute genetically distinct populations. To test this hypothesis we generated a dataset of more than 5000 unlinked single nucleotide polymorphisms (SNPs) using restriction site associated DNA sequencing (RADSeq) [36] for king penguins from four colonies spread across their range (Fig. 1). We aimed to identify population structure, as well as distinct phylogenetic lineages that may have been associated with past glacial refugia. Previous studies have shown that king penguin numbers were much reduced during the last ice age [37], and the species’ range may have been contracted into refugia at unknown locations [38]. Finally, we aimed to test the hypothesis that the recently founded colony at the Falkland Islands [39] was established via migration from nearby South Georgia (Fig. 1). Throughout we use the term ‘dispersal’ to refer to individual movements away from the natal colony and ‘migration’ to refer to an individual breeding at a different colony from its natal colony.

## Methods

### Sampling

Blood was collected from 16 king penguins at each of: Volunteer Point on the Falkland Islands (Feb 2014), Fortuna Bay on South Georgia (Dec 2012), Baie du Marin on Possession Island in the Crozet Islands (Dec 2003–Jan 2004) and Sandy Bay on Macquarie Island (Dec 2005–Jan 2006) (Fig. 1). To prevent biting and minimize stress during handling [40], king penguins were either seized with both hands and the flippers were restrained with the head placed under the arm of the handler, or they were wrapped in cushioned material to cover the head and prevent movement. A second handler took 1 mL blood from the brachial or ulnar vein using a 25G or 23G needle and 1 mL syringe, after cleaning the area with an alcohol swab. Total restraint time was generally two to three minutes. All field activities were conducted under permits from the Falkland Islands Government, the Government of South Georgia and the South Sandwich Islands and the Tasmanian Parks Department, and also received ethical approval from the University of Oxford, the University of Western Australia, the Auburn University Institutional Animal Care and Use Committee and the Institut Polaire P. E. Victor. Blood samples were transported to the UK at ambient temperature in RNAlater (Life Technologies) or in Queen’s Lysis buffer, and stored at −20 °C or −80 °C until extraction.

### Sequencing

DNA was extracted from the 64 blood samples using a QIAGEN DNeasy Blood and Tissue Kit following the manufacturer’s protocol, but modified to include 40 μL proteinase K at the digestion step and with the incubation time extended to 3 h. The samples were treated with 1 μL Riboshredder (Epicentre) to reduce RNA contamination. DNA concentration was measured with a Qubit (ThermoFisher Scientific) and high molecular weight was confirmed on a 1 % gel. We sequenced the mitochondrial hypervariable region (HVR; 620 base pairs; GenBank accessions: KX857217-KX857259) because this marker has revealed phylogeographic patterns within other penguin species [28, 29, 41]. The HVR was amplified in all samples using primers F-0225 (5’-GGAACCTCCCAAAGAGTACCA) and R-INR (5’-CCAACCAGATGTATCGGTGA) [28]. PCR products were sequenced using the Sanger method by Macrogen Europe. Geneious v5.5.9 was used for alignment.

We employed RADSeq to generate a dataset of genome-wide SNPs to assess population structure among the king penguin colonies. RAD libraries were prepared using the SbfI restriction enzyme, which was chosen because it produces a large number of RAD loci in king penguins [37]. RADSeq for all individuals was performed at the Edinburgh Genomics Facility, University of Edinburgh (https://genomics.ed.ac.uk/) as described in Gonen et al. [42] after Etter et al. [43]. Briefly, 250 ng of DNA per individual was digested with SbfI-HF (NEB), followed by ligation to barcoded P1 adapters. The uniquely barcoded individuals were pooled into multiplexed libraries, and each library sheared into fragments of ∼ 300—400 bp. Fragments were size selected using gel electrophoresis. The libraries were blunt ended (NEB Quick Blunting Kit) and A-tailed prior to ligation with P2 adapters (IDT). Enrichment PCR was performed to increase yield, followed by product purification with Ampure beads. The pooled, enriched libraries were checked for size and quantity using Qubit and a qPCR assay. Each library was then sequenced in a lane of the Illumina HiSeq 2500 using 125 base paired-end reads in high output mode (v4 chemistry).

### Bioinformatics

FastQC was used to assess read quality and check for adapter contamination. We used *process_radtags* within the Stacks pipeline v1.35 [44, 45] to de-multiplex, trim and clean reads. We then truncated reads to 113 bp to exclude the four terminal bases in order to avoid poor sequence quality. We excluded read pairs in which either read had uncalled bases, a low quality score and/or a barcode or cut-site with more than one mismatch. The remaining paired reads were aligned to the emperor penguin reference genome (http://gigadb.org/dataset/100005) using *bwa-mem* [46]. We prevented terminal alignments by enforcing a clipping penalty of 100. Reads with more than five mismatches, multiple alignments and/or more than two indels were removed using a custom python script (filter.py, available online [47]). We removed PCR duplicates with Picardtools (http://broadinstitute.github.io/picard).

We used the Stacks pipeline (*pstacks – cstacks – sstacks – rxstacks – cstacks – sstacks - populations*) to prepare a dataset of unlinked, filtered SNPs from the RAD reads, following many of the suggestions outlined in the framework of Benestan et al. [48]. In *pstacks* we selected a minimum stack depth of six reads mapping to the same location and used the bounded SNP model with a significance level of α = 0.05, an upper bound of 0.1 and a lower bound of 0.0041 (corresponding to the highest sequencing error rate recorded by phiX spikes in the sequencing lanes). All 64 individuals were used to build the catalog in *cstacks*. In *rxstacks* we removed confounded loci (those with a biologically implausible number of haplotypes, such as from repetitive sequences or paralogous loci) with a conservative confidence limit of 0.25. Also in *rxstacks*, we removed excess haplotypes from individuals as well as any loci with a mean log likelihood < −10. Further filtering was conducted in the *populations* module. We removed SNPs with a minor allele frequency (MAF) < 0.01 because these are likely to be the result of sequencing errors. We also removed loci with a heterozygosity > 0.5, as these could be paralogs [48]. A single SNP per RAD-tag was chosen at random in order to remove tightly linked SNPs from the dataset. We also specified that a locus must be present in all colonies to be included in the final dataset, as well as genotyped in at least 80 % of individuals from each colony. We then removed any SNPs with a mean coverage exceeding 100X using vcftools v0.1.13 [49] to avoid SNPs from repetitive regions of the genome. We also removed SNPs that were out of Hardy Weinberg equilibrium (HWE) in > 50 % of the colonies when *p* < 0.01 using the *adegenet* package in R [50, 51] and vcftools. Finally, PGDSpider v2.0.8.2 [52] was used to convert the vcf file into other formats for subsequent analyses.

### Outlier loci detection

We investigated whether SNPs were potentially under selection before proceeding with population genetic analyses, because loci under either directional or balancing selection violate the assumption of neutrality that is a caveat of most population genetic methods. We used a Bayesian *F*_*ST*_ outlier test as implemented in BayeScan 2.1 [53] to identify loci to be discarded from the neutral dataset. BayeScan has been shown to have good power for detecting loci genuinely under selection under a range of demographic scenarios, but with an accompanying high false-positive rate [54]. Given that our reason for testing for outlier loci was to obtain a truly neutral dataset, we are not concerned by the high false-positive rate in this case. We set the prior odds of neutrality parameter at five, which refers to the probability that a given locus in the dataset is under selection (i.e. for every five loci one is under selection). This prior was chosen as we aimed to remove all loci that could possibly be under selection. We deemed *q*-values of < 0.1 to be a significant result, meaning that for a dataset of 100 *F*_*ST*_ outliers we can expect ten of these to be false-positive neutral loci [54, 55].

### Contemporary population structure

The genetic structuring among king penguin colonies was assessed using several different methods. Firstly, the Weir and Cockerham [56] unbiased estimator of *F*_*ST*_ was calculated between all pairs of colonies using Genodive v2.0b27 [57]. The hypothesis of departure from panmixia was tested with 5000 random permutations of the data to determine the statistical significance of each pairwise *F*_*ST*_ value between colonies, with the significance level adjusted for multiple testing using Sequential Goodness of Fit (SGoF+) [58].

To identify the number of genetic populations (“clusters”) among the 64 individuals, we used the *find.clusters K*-means clustering algorithm within the *adegenet* package [50, 51], retaining all principal components. We also used a Bayesian clustering approach with a Markov chain Monte Carlo (MCMC) sampling procedure within *structure* v2.3.4 [59]. The analysis estimated the membership coefficient of each individual to each of the inferred clusters, effectively assigning individuals to genetic populations. We used the admixture model with correlated allele frequencies, because our pairwise *F*_*ST*_ results suggested that it is highly likely that these colonies have experienced admixture in the past and/or are still exchanging migrants. Models were run both with and without location priors to reflect the colony that each individual was sampled at, to detect subtle versus strong population structure. We conducted an initial run to infer the value of lambda, using a setting of *K* = 1 and an MCMC length of 100,000 generations (with the first 50,000 discarded as burn-in), allowing lambda to vary. The value of lambda was then fixed at 0.39 for subsequent analyses. *K* values (the number of inferred clusters) from one to four were tested, with each value of *K* run a total of ten times from different random seeds. Each analysis was run for 150,000 generations with the first 50,000 discarded as burn-in. *structure harvester* web v0.6.94 [60] was used to compare *K* values using the Evanno method [61] and prepare files for CLUMPP [62]. Replicate runs for each value of *K* were aligned using CLUMPP to check for multimodality, and the membership coefficients of each individual to each cluster were visualised with DISTRUCT v1.1 [63].

Discriminant Analysis of Principal Components (DAPC) [64] can be used to describe clusters in genetic data by creating synthetic variables (discriminant functions) that maximise variance among groups whilst minimising variance within groups. DAPC was run when individuals were grouped by colony of origin and when individuals were grouped by the genetic clusters found in our other analyses, for comparison. These groups were (1) South Georgia and (2) the Falkland Islands, Crozet and Macquarie. The optimal number of principal components (PCs) to retain in each analysis was determined by the average of 20 runs of the function *optim.a.score*.

We conducted individual-based population assignment tests, in which an assignment algorithm attempts to assign the individuals in the test set to their population of origin [65]. Individuals were grouped into the two genetic populations we described above. As assignment tests can be sensitive to uneven sample numbers, we randomly sampled 16 individuals from the larger population to match the size of the South Georgia population. Each group was divided into a *training* set and a *hold*-*out* set and we identified the most informative SNPs for colony assignment using the *training* set in TRES v1.0 [66]. We used the Informative for Assignment test (I_n_) to identify ancestry informative markers (AIMs), as I_n_ has been shown to be the most powerful method for estimating ancestry proportions [67]. For population assignment tests it is recommended to trial different numbers of SNPs, therefore, we exported the top 100, 200, 500, 1000 and 2000 most informative SNPs. These SNP datasets were used to assign the *hold-out* set of individuals to their populations of origin within Genodive. If the minor allele was not sampled in either population (i.e. its frequency was zero) the frequency was replaced with 0.005 as recommended by Paetkau et al. [68]. We used the likelihood that the individual comes from the population it was sampled in (*L*_*h*_) as the test statistic and a Monte Carlo test with 10,000 generations to estimate the null distribution of likelihood values. The threshold value was defined for each population based on the null distribution, at α = 0.05.

### Past population patterns

We used a species tree approach, as implemented in SNAPP [69] within BEAST v2.4.0 [70], to estimate the evolutionary relationships and order of splitting among the geographically isolated colonies to determine whether any of the colonies may have been glacial refugia in the past, as well as the source of the new Falkland Islands colony. SNAPP uses a coalescent method to infer species trees from unlinked biallelic markers, such as SNPs. SNAPP is highly computationally demanding and analysis of the full dataset of individuals was implausible. We therefore selected two representative individuals from each colony (i.e. four haplotypes) for analysis, and to ensure consistency of the posterior we ran the analysis twice with different randomly-selected colony representatives. Any SNPs that were no longer polymorphic within the reduced datasets were removed from analysis, leaving datasets of 2668 and 2626 SNPs. The mutation rates (u and v) were calculated from the data, rather than estimated as part of the MCMC. We ran the MCMC for 5 million generations with a burn-in of 10 %. This was more than sufficient for convergence, with Tracer v1.6 [71] indicating ESSs > 4000. The likelihood plots were also visually inspected for convergence. The Bayesian method results in not a single topology, but a posterior distribution of the possible topologies; we used DensiTree v2.0.1 [72] to visualise the entire posterior distributions of trees as a cloudogram, excluding a 10 % burn-in.

We used RAxML v8.2.7 [73] to infer maximum likelihood (ML) phylogenies among the full dataset of king penguin individuals. We applied an ascertainment bias correction to the likelihood calculations, as recommended when using SNPs to account for the lack of invariant sites [74]. For the ascertainment correction to function, all invariant sites must be removed. In practice, this means that an alignment site consisting of only heterozygotes and homozygotes for a single allele (e.g. an alignment site that is only Rs and As with no Gs) is considered potentially invariant by RAxML and must be removed. We filtered out such sites using the Phrynomics R script (https://rstudio.stat.washington.edu/shiny/phrynomics/). After this filtering step 1727 SNPs remained in the dataset. We conducted a rapid bootstrap analysis and search for the best-scoring maximum likelihood tree in a single program run using the MRE-based bootstopping criterion [75] to ascertain when sufficient bootstrap replicates had been generated. All searches were conducted under the GTRGAMMA nucleotide substitution model. We also conducted a ML search on the HVR sequences, because HVR has been shown to resolve distinct phylogenetic lineages within Adélie penguins [29, 41, 76], emperor penguins [28] and gentoo penguins [31, 41]. We used the same search protocol as for the SNP dataset, but without an ascertainment bias correction. Finally, we constructed a median-joining haplotype network for the HVR sequences using PopArt (http://popart.otago.ac.nz).

## Results

### Genotyping

The 64 king penguin samples yielded 6.27–55.9 million unpaired reads per individual, with an average of 15.7 million reads per individual. On average, 97.3 % of reads per individual passed the quality filters in *process_radtags* and, of these, an average of 97.7 % successfully aligned to the emperor penguin reference genome. After specifying a minimum stack depth of six, a total of 34,171 RAD-tags remained, containing 35,766 SNPs. Our SNP filtering protocols resulted in a final dataset of 5154 SNPs [47] for use in subsequent analyses. Of these we detected no loci that were putatively under selection (BayeScan output available online [47]) and none that were out of HWE in > 50 % of colonies. There were no notable differences in genetic diversity measures (number of private alleles, expected heterozygosity, observed heterozygosity or nucleotide diversity) among colonies (Table 1).

**Table 1:** Genetic diversity measures by colony, based on variant (SNP) sites only.

	*N* private alleles	*H*_E_ (mean)	*H*_E_ (Var)	*H*_E_ (StdErr)	*H*_O_ (mean)	*H*_O_ (Var)	*H*_O_ (StdErr)	π (mean)	π (Var)	π (StdErr)
Falklands	148	0.1179	0.0175	0.0018	0.1107	0.0170	0.0018	0.1219	0.0187	0.0019
South Georgia	147	0.1161	0.0174	0.0018	0.1066	0.0161	0.0018	0.1200	0.0185	0.0019
Crozet	117	0.1178	0.0177	0.0019	0.1151	0.0183	0.0019	0.1217	0.0189	0.0019
Macquarie	180	0.1187	0.0178	0.0019	0.1115	0.0175	0.0018	0.1225	0.0189	0.0019

Number of private alleles, expected heterozygosity (*H*_E_), observed heterozygosity (*H*_O_) and nucleotide diversity (π)

### Genetic populations of king penguins

**Fig. 2:**
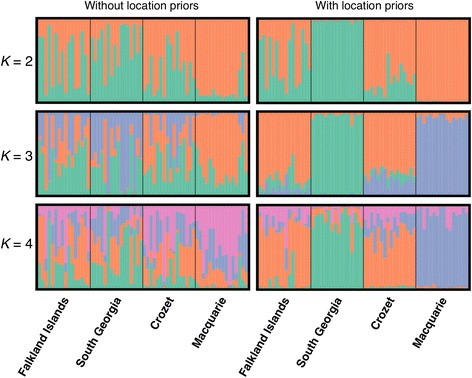
Population assignment of individuals by Bayesian clustering in *structure*. Membership coefficients for each individual are shown by vertical bars with the clusters represented by colours. The Evanno method selected *K* = 2 when no location prior was used and *K* = 3 when a location prior was used. When *K* = 3 the three clusters correspond to 1) the Falkland Islands and Crozet colonies, 2) the South Georgia colony, and 3) the Macquarie Island colony

We conducted multiple analyses of population assignment and delimitation to identify the number and geographic boundaries of distinct genetic populations among the four sampled king penguin colonies. The optimal number of clusters among the 64 individuals were *K* = 3 and *K* = 2 for *structure* analyses with and without location priors, respectively, as determined by the Evanno method. However, the highest posterior mean log probability of the data for both scenarios (i.e. with and without the sampling location specified as a prior) was at *K* = 1. The rate of change in log probability (delta*K*) is not defined at *K* = 1, and so the Evanno method is unable to determine whether this is actually the true value of *K*. This suggests that the signal for multiple clusters is weak. Inspection of the individual assignment plots (Fig. 2) showed that three clusters explain the majority of the subtle structure. The Falkland Islands and Crozet Islands cluster together, whereas the Macquarie Island and South Georgia colonies appear differentiated. The *K*-means clustering algorithm was unable to distinguish these clusters as the lowest value of the BIC, which indicates the optimal clustering solution, was found at *K* = 1.

Our measures of pairwise *F*_*ST*_ (Table 2) indicate that the Crozet and Falkland Islands colonies are not differentiated from one another (*F*_*ST*_ = −0.001), and that Macquarie and Crozet Islands are not significantly differentiated from each other (*F*_*ST*_ = 0.001). All other pairs of populations are statistically significantly differentiated after SGoF+ correction for multiple tests, however, the values of *F*_*ST*_ are very small (0.003–0.005), indicating only subtle genetic differences between these pairs of colonies. Therefore there are at least two slightly differentiated genetic populations among the sampled colonies: (1) the South Georgia population and (2) a population including the Falkland Islands, Crozet and Macquarie.

**Table 2:** Pairwise genetic differentiation (*F*_*ST*_) between pairs of colonies

	Falkland Islands	South Georgia	Crozet
South Georgia	0.003*		
Crozet	−0.001	0.003*	
Macquarie	0.003*	0.005*	0.001

Results that are significantly different from zero at the α = 0.05 level, following SGoF+ correction, are indicated with asterisks

**Fig. 3:**
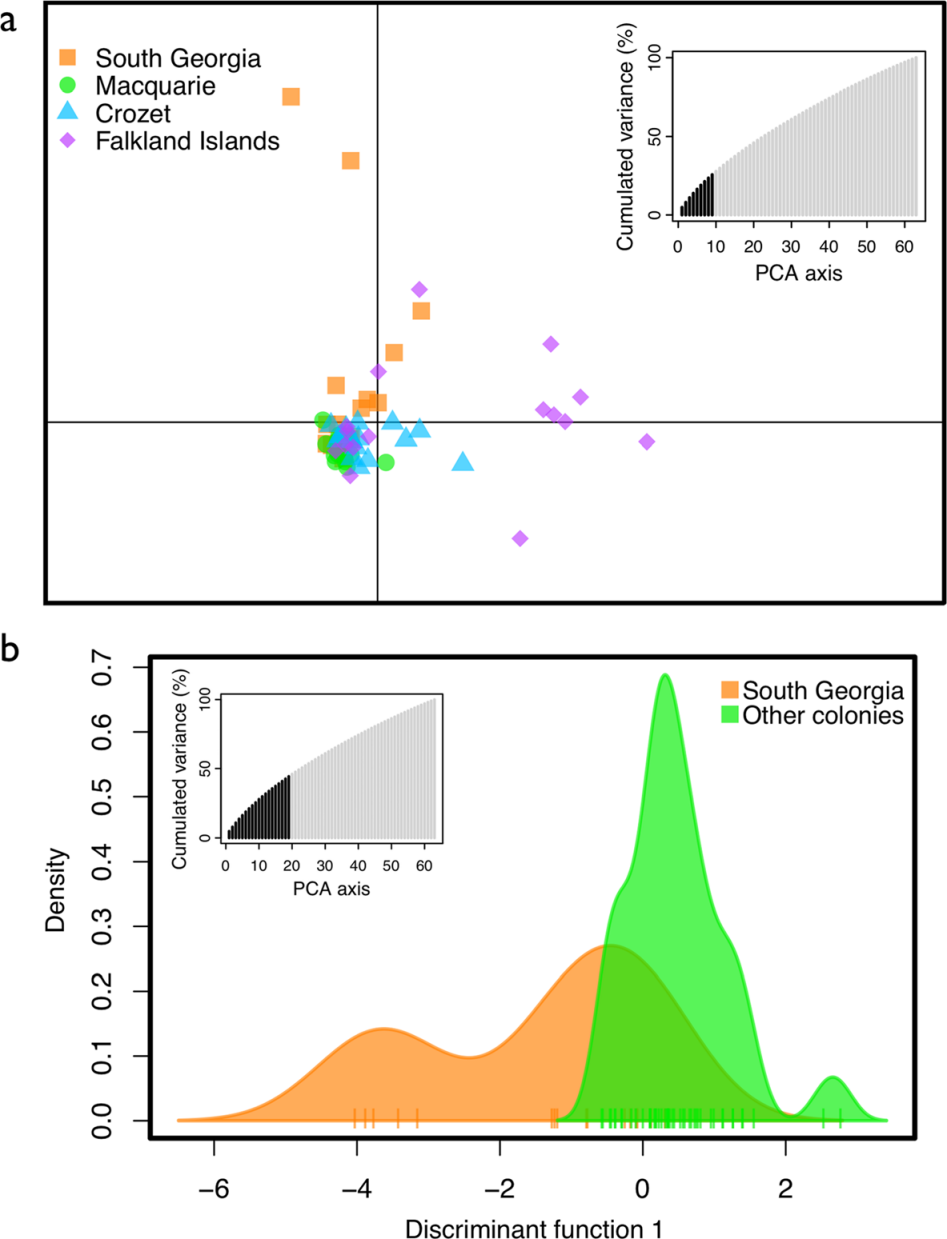
Discriminant analysis of principal components. Individuals are grouped by **a**) their colony of origin and **b**) the two genetic clusters identified by other analyses. The retained PCs are shown in *black* on the inset graphs

DAPC was unable to distinguish among the four sampled colonies or between the two slightly differentiated populations, with the distribution of individuals overlapping in both scenarios (Fig. 3). For the individual-based population assignment tests, the 100 SNP dataset was found to be best at assigning the test set of individuals back to their population of origin. However, the test performed poorly, with only seven individuals assigned correctly out of the 16 individuals in the test dataset. Given that there were only two possible populations of origin, this is slightly worse than assigning individuals to colonies at random. This again suggests that there is very little differentiation among the king penguin colonies.

Overall, our analyses of population structure among the four king penguin colonies have yielded some surprising results. Despite separation of thousands of kilometers, there is very little genetic differentiation among these colonies. The South Georgia population was subtly differentiated from all other colonies, and the Macquarie population was further very subtly differentiated from some colonies by a subset of our analyses. It is particularly interesting that the Falkland Islands colony is genetically indistinguishable from the Crozet Islands colony, despite a separation of *ca*. 7500 km, whereas the nearby South Georgia colony is differentiated; based on our results it seems most likely that the Falkland Islands colony was founded by individuals from the Crozet Islands, rather than nearby South Georgia, even though there seems to be no obvious biological explanation for why this might be so.

### Phylogeography

**Fig. 4:**
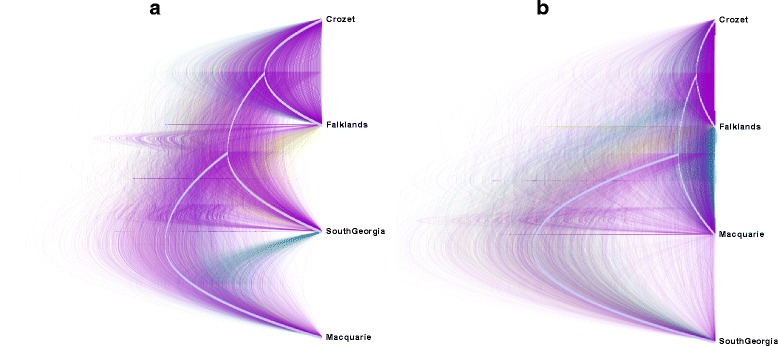
Evolutionary relationships among colonies. The full posterior distributions of trees from the SNAPP analyses, excluding a 10 % burn-in, are shown. The colours represent the different topologies; *purple* is the most highly supported, *teal* is the next most supported, and *gold* is the least supported. The consensus tree is shown in *grey*. (**a**) and (**b**) are the outcomes of the two different analyses with different randomly selected representative individuals

We attempted to ascertain the branching structure among colonies using the species tree approach implemented in SNAPP. We have presented the full posterior distribution of trees in order to highlight the uncertainty in the topology (Fig. 4). The majority of the topologies support the grouping of the Falkland and Crozet Islands colonies (Fig. 4), congruent with our *structure* and pairwise *F*_*ST*_ results. However, aside from this one clade, the rest of the branching structure among the colonies is unresolved.

We constructed maximum likelihood phylogenies for the full set of individuals using both HVR and the dataset of SNPs in order to determine if there are any strongly supported phylogenetic lineages that are not necessarily affiliated with the contemporary colony sites. The MRE bootstopping-criterion was satisfied by 550 and 800 bootstraps for the SNP and HVR searches, respectively. The best-scoring likelihood and majority rule extended consensus trees for the SNP dataset had very low support across the entire topology, with only a single node having a branch support value > 50 (topology not shown). The HVR topology did not show any more resolution, with 75 % of nodes in the tree having branch support values < 50 and no evidence of any well-supported phylogenetic lineages (topology not shown). A median joining network of the haplotypes of the mitochondrial HVR also showed no clear phylogeographic pattern and no evidence of ancestral haplotypes (Additional file 1: Figure S1). Overall, there are no distinct lineages among king penguins, no remnant signatures of refugia and no evidence for the order of colonisation of the islands.

## Discussion

In the first study of king penguin global population structure we found very low levels of population differentiation across the species’ entire distribution, despite using 5154 SNPs distributed throughout the genome. Penguins from the Crozet Islands were not genetically differentiated from those 7450 km west on the Falkland Islands, nor those 7100 km east on Macquarie Island. There was very low, yet statistically significant, genetic differentiation between the colony on South Georgia and all other colonies, including the Falkland Islands located only 1400 km to the northwest. Our phylogeographic analyses showed no evidence of distinct king penguin lineages.

The lack of genetic differentiation across such vast distances is surprising given that king penguin colonies are sparsely distributed across the Southern Ocean. There are very few locations that support king penguin breeding between the archipelagos we have sampled; the only other colonies are in the Indian Ocean sector close to the Crozet Islands (Fig. 1). Therefore, there are very few “stepping stones” between colonies and the lack of differentiation between Crozet and Macquarie suggests that migration is not distance-limited.

There are two alternative explanations for the observed low levels of genetic differentiation among king penguin colonies. Firstly, it could be the result of frequent migration of individuals among these isolated archipelagos. In this scenario, dispersing individuals must also be recruited into the breeding population upon arrival, if they are to contribute to the gene flow that is maintaining near genetic homogeneity of king penguins. Alternatively, all extant colonies may share a common ancestral population and insufficient time has passed for them to diverge, even if they are now isolated. Despite the large geographic distances separating them, there is a growing body of evidence to suggest that king penguin colonies do exchange migrants [23–25]; we therefore consider the former hypothesis, that migration is maintaining gene flow among populations, to be the most likely explanation for the genetic similarity found here.

The recent formation of new colonies at Volunteer Point on the Falkland Islands [39], Possession Island in the Crozet Islands [11] and on Macquarie Island [9] provides direct evidence that some individuals will breed away from their natal colony. A handful of individuals banded as breeders have also been observed breeding at non-natal colonies within the Crozet Islands (Bost, C. A. *pers comm*). Furthermore, the rate of population growth at Possession Island over the past several decades has been too great to have been maintained by intrinsic recruitment alone; therefore, the population growth must be partially attributable to immigration [11]. Small numbers of king penguins, and in particular juveniles, have been observed at colonies up to 5600 km from their natal colonies [23–25, 77]. This suggests that king penguins probably prospect other colonies and breeding habitats, including those far from their natal colony, and this may occur most often before they begin to breed. This prospecting behavior may facilitate emigration when conditions at the natal colony are less favorable than those found elsewhere.

Previous studies have shown that seabirds with large foraging ranges or those that disperse widely in the non-breeding season are least likely to show genetic differentiation among colonies [3]. During the summer breeding season, king penguin foraging trips typically last days to weeks and can cover hundreds to thousands of kilometers [78]. During the winter, king penguins rarely provision their chicks, and so adults are not restricted to central-place foraging. These winter foraging trips often take them over 1500 km away from their colonies to the marginal ice zone around Antarctica, and journeys in excess of 10,000 km have been recorded, although there is no evidence for foraging range overlap among breeding colonies thus far [79, 80]. The few juveniles that have been tracked after fledging dispersed widely in their first 6 months, probably bringing them into contact with individuals from other colonies [81]. Therefore juvenile dispersal and possibly also foraging range overlap during the non-breeding season appears to facilitate gene flow in king penguins, as it does in a variety of seabirds [4], but without more data on the winter dispersal of king penguins it is difficult to determine the relative importance of these mechanisms.

It is unclear whether the observed low level of genetic differentiation is maintained by consistent background levels of migration, or whether episodic periods of higher migration have occurred, or both. Abiotic factors such as glacial expansion and retreat, landslides, erosion, flooding, volcanic activity or other such catastrophic events [9] could result in periods of increased emigration, whilst large-scale climatic anomalies that affect the proximity of oceanic fronts and prey availability to colonies [7] could also increase the emigration rate if adults perceive the habitat quality to have declined. The harvesting of king penguins during the late 19th and early 20th century could have temporarily increased emigration rates, if individuals emigrated to less disturbed colonies. Biotic factors could also play a role, as emigration may be favored when colonies reach carrying capacity and/or density-dependent factors limit population growth, such as competition for food and nest sites, predation and pathogen load [11]. The colony at Lusitania Bay on Macquarie Island is thought to have reached carrying capacity in 1975 when all available breeding habitat was occupied and individuals were forced to spill over to other colonies [9, 10]. Two large colonies, Petite Manchotière and Jardin Japonais, on Possession Island in the Crozet Islands are also believed to have reached carrying capacity in the late 1980s, with all areas free of vegetation being occupied [11]. As these colonies approached carrying capacity, the formation of the two new colonies on Possession Island in 1979 and 1986 could have been the direct result of these large colonies spilling over, with individuals emigrating rather than competing for nest spaces at their natal colonies. This could also account for the colonization of the Falkland Islands in the late 1970s. We found no evidence for genetic differentiation between the Falkland Islands and the Crozet Islands, and the colonies grouped together in our species tree analysis. Therefore it seems likely that individuals from the Crozet Islands, possibly forced to emigrate due to competition for space at their natal colonies, founded the population at the Falkland Islands. This finding was somewhat unexpected given the 7450 km between the populations, and the relative proximity of the South Georgia population just 1400 km away. Furthermore, the observation of an individual that was banded as a chick in South Georgia but was later found breeding in the Falkland Islands [82] would also tend to suggest that the Falkland Island population would have been founded by immigrants from South Georgia. However, our genetic results indicate that there has been a higher rate of immigration from the Crozet Islands than from South Georgia.

The difference in the oceanic regime experienced by king penguins at South Georgia could explain why this colony was genetically differentiated from all other colonies [4]. South Georgia lies to the south of the Polar Front, whilst all other studied colonies lie to the north, and thus birds at South Georgia experience colder oceanic and air temperatures and a more krill-dominated food web. The different ecological conditions either side of the Polar Front appear to act as a barrier to gene flow in many species [83], including gentoo penguins [41], although this effect appears much weaker in king penguins.

While it would be useful to be able to determine the actual migration rates among the colonies studied here, the very low levels of genetic differentiation preclude the calculation of accurate estimates. Hence, whether the colonies are demographically linked or should be considered as separate management units cannot be determined [84]. Furthermore, there is currently no generalized framework for determining the level of migration necessary to maintain demographic linkage [85]. BayesAss [86], which is typically used to determine recent directional migration rates between populations (gene flow occurring over the last few generations), has been found to be unreliable when *F*_*ST*_ values are less than 0.05 (i.e. an order of magnitude greater than observed among king penguins) [87]. Methods to estimate migration based on *F*-statistics are also unreliable because the assumptions of the island model [88] that relates *F*_*ST*_ to the number of migrants entering a population (Nm) are usually violated in natural systems, limiting the amount of quantitative information about migration that can be gained from *F*-statistics [89]. Finally, coalescent methods, such as Migrate-n [90], which estimate migration over evolutionary timescales, are also likely to be inaccurate when population differentiation is low and only a small number of loci can be used because of massive computational demands [27]. Coalescent methods also rely on an estimate of the mutation rate for the specific loci used in the analysis, to translate the mutation-scaled migration rate into an estimate of the number of migrants entering a population, and accurate mutation rates are difficult to estimate for RAD loci [91, 92].

The lack of phylogenetic signal or mitochondrial lineages suggests that small populations of king penguins have not been isolated from one another in their recent history. Some colonies went through rapid declines when king penguins were harvested for their blubber. For example, the Macquarie Island colony was reduced from hundreds of thousands of birds to about 3000 [9]. These rapid declines, although extreme demographically, were unlikely to have caused a genetic bottleneck resulting in lineage divergence, as they were neither severe enough nor lasted long enough for significant genetic drift to have taken place. Certainly there is no signature of recent genetic bottlenecks in our data. Furthermore, if the harvesting also caused a pulse of increased emigration and gene flow, then genetic diversity is unlikely to have been affected. Indeed, the Macquarie Island population appears to have retained genetic diversity throughout the period of harvesting, as demonstrated by a comparison of ancient, pre-harvest genetic diversity to the modern population [93]. The king penguin population at La Baie du Marin colony on the Crozet Islands was much smaller during the last glacial maximum (LGM), and then rapidly increased in size following Holocene warming [37]. LGM conditions appear to have isolated refugial populations of Adélie [29, 41, 76], emperor [28] and gentoo penguins [31, 41] in ice age refugia, resulting in distinct mitochondrial lineages. Our results do not support this for king penguins, although distinct lineages could exist outside of the colonies we sampled. The single mitochondrial lineage found here suggests that gene flow between populations of king penguins was maintained during the LGM even if their population sizes were reduced, and their tendency to disperse probably allowed this. Interestingly, the emperor penguin, the sister-species to king penguins in the *Aptenodytes* genus, also has remarkable dispersal abilities, exhibiting very low levels of genetic differentiation around its global range [27, 28], similar to Adélie penguins [30, 41, 76]. Yet we see distinct mitochondrial lineages in the emperor penguin, with origins dated to the last ice age [28], that are not apparent in king penguins. We propose that the sub-Antarctic distribution of king penguins may explain this contrast. Many of the sub-Antarctic islands king penguins breed on have been heavily glaciated [94], reducing available breeding area, but the increased sea ice extent during glacial periods [95] would probably not have created barriers to king penguin migration as it did not extend as far north as the king penguin’s sub-Antarctic range.

## Conclusions

Our study has revealed an unexpectedly low level of genetic differentiation among king penguin colonies spanning thousands of kilometers of the Southern Ocean, with some colonies separated by more than 7000 km showing no significant genetic divergence. On the other hand, the South Georgia colony does appear to be subtly differentiated from all other studied colonies, despite it lying in close proximity to the Falkland Island colony.

The very low level of genetic differentiation we have shown among king penguin colonies needs to be considered in management plans to mitigate future climate change impacts on the species. Colonies within the same archipelago are highly likely to be panmictic and demographically linked, and thus monitoring of king penguins should be considered at the archipelago level, rather than at the colony level. The subtle differentiation we found between some archipelagos, and our inability to determine whether migration is consistent or episodic, cautions against the assumption that colonies are demographically linked globally. Therefore, as a precaution, we recommend that populations at the archipelago level are managed as separate units. Given the relatively few archipelagos that host king penguins, and that climate change effects will be heterogeneous across their range, declines at any of these locations should be considered as significant and would hinder the recovery of the species, even if a loss of genetic diversity would not occur.

Demographic models that attempt to forecast extinction risk in response to large-scale climate change must also take into account migration. Recently, Tavecchia et al. (2016) showed that migration can decouple the relationship between population growth rates and climate variables, such that even if demographic rates are sensitive to climate-driven variations, this does not necessarily result in climate-driven population changes when immigration of new individuals occurs [96]. Migration could therefore buffer king penguins against their forecasted risk of extinction under climate change [6] although it may not protect them completely [7].

## Additional file

Additional file 1: Figure S1. Median-joining haplotype network of king penguin HVR sequences. (PDF 281 kb)

## References

[1] Manel S, Schwartz MK, Luikart G, Taberlet P (2003). Landscape genetics: combining landscape ecology and population genetics. TREE.

[2] Walther G-R, Post E, Convey P, Menzel A, Parmesan C, Beebee TJ, Fromentin J-M, Hoegh-Guldberg O, Bairlein F (2002). Ecological responses to recent climate change. Nature.

[3] Friesen VL, Burg TM, McCoy KD (2007). Mechanisms of population differentiation in seabirds. Mol Ecol.

[4] Friesen VL (2015). Speciation in seabirds: why are there so many species… why aren’t there more?. J Ornithol.

[5] Coulson J, Schreiber EA, Burger J (2002). Colonial breeding in seabirds. Biology of marine birds.

[6] Le Bohec C, Durant JM, Gauthier-Clerc M, Stenseth NC, Park Y-H, Pradel R, Gremillet D, Gendner J-P, Le Maho Y (2008). King penguin population threatened by Southern Ocean warming. PNAS.

[7] Bost CA, Cotté C, Terray P, Barbraud C, Bon C, Delord K, Gimenez O, Handrich Y, Naito Y, Guinet C (2015). Large-scale climatic anomalies affect marine predator foraging behaviour and demography. Nat Commun.

[8] Bost C, Delord K, Barbraud C, Cotté C, Péron C, Weimerskirch H, Borboroglu PG, Boersma PD (2013). King Penguin. Penguins: Natural History and Conservation.

[9] Van Den Hoff J, McMahon CR, Field I (2009). Tipping back the balance: recolonization of the Macquarie Island isthmus by king penguins (Aptenodytes patagonicus) following extermination for human gain. Antarc Sci.

[10] Rounsevell D, Copson G (1982). Growth rate and recovery of a king penguin, aptenodytes patagonicus, population after exploitation. Wildlife Res.

[11] Delord K, Barbraud C, Weimerskirch H (2004). Long-term trends in the population size of king penguins at Crozet archipelago: environmental variability and density dependence?. Polar Biol.

[12] Conroy J, White M (1973). The breeding status of the king penguin (aptenodytes patagonica). Bull Brit Antarct Survey.

[13] IUCN (2016). The IUCN Red list of threatened species.

[14] Sokolov S, Rintoul SR, Wienecke B. Tracking the Polar Front south of New Zealand using penguin dive data. Deep-Sea Res Pt 1. 2006; 53(4):591–607

[15] Charrassin J-B, Bost C-A (2001). Utilisation of the oceanic habitat by king penguins over the annual cycle. Mar Ecol Prog Ser.

[16] Scheffer A, Bost C-A, Trathan PN (2012). Frontal zones, temperature gradient and depth characterize the foraging habitat of king penguins at South Georgia. Mar Ecol Prog Ser.

[17] Bost C-A, Cotté C, Bailleul F, Cherel Y, Charrassin J-B, Guinet C, Ainley DG, Weimerskirch H (2009). The importance of oceanographic fronts to marine birds and mammals of the southern oceans. J Mar Syst.

[18] Jouventin P, Capdeville D, Cuenot-Chaillet F, Boiteau C (1994). Exploitation of pelagic resources by a non-flying seabird: satellite tracking of the king penguin throughout the breeding cycle. Mar Ecol Prog Ser.

[19] Péron C, Weimerskirch H, Bost C-A (2012). Projected poleward shift of king penguins’ (aptenodytes patagonicus) foraging range at the Crozet Islands, southern Indian ocean. P Roy Soc Lond B Biol.

[20] Saraux C, Viblanc VA, Hanuise N, Le Maho Y, Le Bohec C (2011). Effects of individual Pre-fledging traits and environmental conditions on return patterns in juvenile king penguins. PLoS One.

[21] Wright S (1969). Evolution and the genetics of populations: Vol. 2. The theory of gene frequencies.

[22] Baylis AM, Orben RA, Pistorius P, Brickle P, Staniland I, Ratcliffe N (2015). Winter foraging site fidelity of king penguins breeding at the Falkland Islands. Mar Biol.

[23] Gartshore N, Cooper J, Hunter S (1988). Bird ringing at Marion and Prince Edward Islands, 1982–1987; with an analysis of movements since 1951. S Afr J Antarc Res.

[24] Weimerskirch H, Jouventin P, Mougin J, Stahl J, Van BM (1985). Banding recoveries and the dispersal of seabirds breeding in French Austral and Antarctic Territories. Emu.

[25] Woehler EJ (1989). Resighting and recoveries of banded seabirds at Heard Island, 1985–1988. Corella.

[26] Boileau MG, Hebert PD, Schwartz SS (1992). Non-equilibrium gene frequency divergence: persistent founder effects in natural populations. J Evol Biol.

[27] Cristofari R, Bertorelle G, Ancel A, Benazzo A, Le Maho Y, Ponganis PJ, Stenseth NC, Trathan PN, Whittington JD, Zanetti E (2016). Full circumpolar migration ensures evolutionary unity in the emperor penguin. Nat Commun.

[28] Younger JL, Clucas GV, Kooyman G, Wienecke B, Rogers AD, Trathan PN, Hart T, Miller KJ (2015). Too much of a good thing: sea ice extent may have forced emperor penguins into refugia during the last glacial maximum. Glob Change Biol.

[29] Ritchie PA, Millar CD, Gibb GC, Baroni C, Lambert DM (2004). Ancient DNA enables timing of the Pleistocene origin and Holocene expansion of two adélie penguin lineages in Antarctica. Mol Biol Evol.

[30] Roeder AD, Marshall RK, Mitchelson AJ, Visagathilagar T, Ritchie PA, Love DR, Pakai TJ, McPartlan HC, Murray ND, Robinson NA (2001). Gene flow on the ice: genetic differentiation among adélie penguin colonies around Antarctica. Mol Ecol.

[31] Levy H, Clucas GV, Rogers AD, Leaché AD, Ciborowski KL, Polito MJ, Lynch HJ, Dunn MJ, Hart T (2016). Population structure and phylogeography of the gentoo penguin (pygoscelis Papua) across the Scotia Arc. Ecol Evol.

[32] Freer JJ, Mable BK, Clucas G, Rogers AD, Polito MJ, Dunn M, Naveen R, Levy H, Hart T (2015). Limited genetic differentiation among chinstrap penguin (pygoscelis Antarctica) colonies in the Scotia Arc and western Antarctic Peninsula. Polar Biol.

[33] Fretwell PT, LaRue MA, Morin P, Kooyman GL, Wienecke B, Ratcliffe N, Fox AJ, Fleming AH, Porter C, Trathan PN (2012). An emperor penguin population estimate: the first global, synoptic survey of a species from space. PLoS One.

[34] Lynch H, LaRue M (2014). First global census of the adélie penguin. Auk.

[35] Orsini L, Vanoverbeke J, Swillen I, Mergeay J, Meester L (2013). Drivers of population genetic differentiation in the wild: isolation by dispersal limitation, isolation by adaptation and isolation by colonization. Mol Ecol.

[36] Baird NA, Etter PD, Atwood TS, Currey MC, Shiver AL, Lewis ZA, Selker EU, Cresko WA, Johnson EA (2008). Rapid SNP discovery and genetic mapping using sequenced RAD markers. PLoS One.

[37] Trucchi E, Gratton P, Whittington JD, Cristofari R, Le Maho Y, Stenseth NC, Le Bohec C (2014). King penguin demography since the last glaciation inferred from genome-wide data. Proc R Soc B.

[38] Younger JL, Emmerson LM, Miller KJ (2016). The influence of historical climate changes on Southern Ocean marine predator populations: a comparative analysis. Glob Change Biol.

[39] Pistorius PA, Baylis A, Crofts S, Pütz K (2012). Population development and historical occurrence of king penguins at the Falkland Islands. Antarc Sci.

[40] Le Maho Y, Karmann H, Briot D, Handrich Y, Robin JP, Mioskowski E, Cherel Y, Farni J (1992). Stress in birds due to routine handling and a technique to avoid it. Am J Physiol.

[41] Clucas GV, Dunn MJ, Dyke G, Emslie SD, Levy H, Naveen R, Polito MJ, Pybus OG, Rogers AD, Hart T (2014). A reversal of fortunes: climate change ‘winners’ and ‘losers’ in Antarctic Peninsula penguins. Sci Rep.

[42] Gonen S, Lowe NR, Cezard T, Gharbi K, Bishop SC, Houston RD (2014). Linkage maps of the Atlantic salmon (Salmo salar) genome derived from RAD sequencing. BMC Genom.

[43] Etter PD, Bassham S, Hohenlohe PA, Johnson EA, Cresko WA, Orgogozo V, Rockman MV (2011). SNP discovery and genotyping for evolutionary genetics using RAD sequencing. Molecular methods for evolutionary genetics.

[44] Catchen J, Amores A, Hohenlohe PA, Cresko WA, Postlethwait JH (2011). Stacks: building and genotyping loci de novo from short-read sequences. G3: genes, genomes. Genetics.

[45] Catchen J, Hohenlohe PA, Bassham S, Amores A, Cresko WA (2013). Stacks: an analysis tool set for population genomics. Mol Ecol.

[46] Li H (2013). Aligning sequence reads, clone sequences and assembly contigs with BWA-MEM. arXiv preprint arXiv:13033997.

[47] Clucas GV, Younger JL, Kao D, Rogers AD, Handley J, Miller GD, Jouventin P, Nolan P, Gharbi K, Miller KJ, Hart T. Data from: Dispersal in the sub-Antarctic: King penguins show remarkably little population genetic differentiation across their range. Data Dryad Repository. 2016. http://dx.doi.org/10.5061/dryad.7c0q8 10.1186/s12862-016-0784-z PMC5062852 27733109

[48] Benestan LM, Ferchaud AL, Hohenlohe PA, Garner BA, Naylor GJ, Baums IB, Schwartz MK, Kelley JL, Luikart G (2016). Conservation genomics of natural and managed populations: building a conceptual and practical framework. Mol Ecol.

[49] Danecek P, Auton A, Abecasis G, Albers CA, Banks E, DePristo MA, Handsaker RE, Lunter G, Marth GT, Sherry ST (2011). The variant call format and VCFtools. Bioinformatics.

[50] Jombart T, Ahmed I (2011). adegenet 1.3-1: new tools for the analysis of genome-wide SNP data. Bioinformatics.

[51] Jombart T (2008). adegenet: a R package for the multivariate analysis of genetic markers. Bioinformatics.

[52] Lischer H, Excoffier L (2012). PGDSpider: an automated data conversion tool for connecting population genetics and genomics programs. Bioinformatics.

[53] Foll M, Gaggiotti O (2008). A genome-scan method to identify selected loci appropriate for both dominant and codominant markers: a Bayesian perspective. Genetics.

[54] Lotterhos KE, Whitlock MC (2014). Evaluation of demographic history and neutral parameterization on the performance of FST outlier tests. Mol Ecol.

[55] Storey JD, Tibshirani R (2003). Statistical significance for genomewide studies. PNAS.

[56] Weir BS, Cockerham CC. Estimating F-statistics for the analysis of population structure. Evolution. 1984;38(No. 6):1358–70. 10.1111/j.1558-5646.1984.tb05657.x 28563791

[57] Meirmans PG, Van Tienderen PH (2004). GENOTYPE and GENODIVE: two programs for the analysis of genetic diversity of asexual organisms. Mol Ecol Notes.

[58] Carvajal-Rodriguez A, de Uña-Alvarez J (2011). Assessing significance in high-throughput experiments by sequential goodness of fit and q-value estimation. PLoS One.

[59] Pritchard JK, Stephens M, Donnelly P (2000). Inference of population structure using multilocus genotype data. Genetics.

[60] Earl DA (2012). STRUCTURE HARVESTER: a website and program for visualizing STRUCTURE output and implementing the Evanno method. Conserv Genet Resour.

[61] Evanno G, Regnaut S, Goudet J (2005). Detecting the number of clusters of individuals using the software STRUCTURE: a simulation study. Mol Ecol.

[62] Jakobsson M, Rosenberg NA (2007). CLUMPP: a cluster matching and permutation program for dealing with label switching and multimodality in analysis of population structure. Bioinformatics.

[63] Rosenberg NA (2004). DISTRUCT: a program for the graphical display of population structure. Mol Ecol Notes.

[64] Jombart T, Devillard S, Balloux F (2010). Discriminant analysis of principal components: a new method for the analysis of genetically structured populations. BMC Genet.

[65] Paetkau D, Calvert W, Stirling I, Strobeck C (1995). Microsatellite analysis of population structure in Canadian polar bears. Mol Ecol.

[66] Kavakiotis I, Triantafyllidis A, Ntelidou D, Alexandri P, Megens H-J, Crooijmans RP, Groenen MA, Tsoumakas G, Vlahavas I (2015). TRES: identification of discriminatory and informative SNPs from population genomic data. J Hered.

[67] Ding L, Wiener H, Abebe T, Altaye M, Go RC, Kercsmar C, Grabowski G, Martin LJ, Hershey GKK, Chakorborty R (2011). Comparison of measures of marker informativeness for ancestry and admixture mapping. BMC Genom.

[68] Paetkau D, Slade R, Burden M, Estoup A (2004). Genetic assignment methods for the direct, real-time estimation of migration rate: a simulation-based exploration of accuracy and power. Mol Ecol.

[69] Bryant D, Bouckaert R, Felsenstein J, Rosenberg NA, RoyChoudhury A (2012). Inferring species trees directly from biallelic genetic markers: bypassing gene trees in a full coalescent analysis. Mol Biol Evol.

[70] Bouckaert R, Heled J, Kühnert D, Vaughan T, Wu C-H, Xie D, Suchard MA, Rambaut A, Drummond AJ (2014). BEAST 2: a software platform for Bayesian evolutionary analysis. PLoS Comput Biol.

[71] Rambaut A, Suchard MA, Xie D, Drummond AJ. Tracer v1.6. 2014. http://beast.bio.ed.ac.uk/Tracer

[72] Bouckaert RR (2010). DensiTree: making sense of sets of phylogenetic trees. Bioinformatics.

[73] Stamatakis A (2014). RAxML version 8: a tool for phylogenetic analysis and post-analysis of large phylogenies. Bioinformatics.

[74] Leaché AD, Banbury BL, Felsenstein J, de Oca AN-M, Stamatakis A (2015). Short tree, long tree, right tree, wrong tree: new acquisition bias corrections for inferring SNP phylogenies. Syst Biol.

[75] Pattengale ND, Alipour M, Bininda-Emonds OR, Moret BM, Stamatakis A (2010). How many bootstrap replicates are necessary?. J Comput Biol.

[76] Younger J, Emmerson L, Southwell C, Lelliott P, Miller K (2015). Proliferation of East Antarctic adélie penguins in response to historical deglaciation. BMC Evol Biol.

[77] Weimerskirch H, Stahl J, Jouventin P (1992). The breeding biology and population dynamics of king penguins aptenodytes patagonica on the Crozet Islands. Ibis.

[78] Ratcliffe N, Trathan P (2011). A review of the diet and at-sea distribution of penguins breeding within the CAMLR convention area. CCAMLR Sci.

[79] Pütz K, Ropert-Coudert Y, Charrassin J, Wilson R (1999). Foraging areas of king penguins aptenodytes patagonicus breeding at possession Island, southern Indian Ocean. Mar Ornithol.

[80] Pütz K (2002). Spatial and temporal variability in the foraging areas of breeding king penguins. Condor.

[81] Pütz K, Trathan PN, Pedrana J, Collins MA, Poncet S, Lüthi B (2014). Post-fledging dispersal of king penguins (aptenodytes patagonicus) from two breeding sites in the South Atlantic. PLoS One.

[82] Otley H, Clausen A, Christie D, Huin N, Pütz K (2007). Breeding patterns of king penguins on the Falkland Islands. Emu.

[83] Rogers AD, Rogers AD, Johnston NM, Murphy EJ, Clarke A (2012). Evolution and biodiversity of Antarctic organisms: a molecular perspective. Antarctic ecosystems: an extreme environment in a changing world.

[84] Palsbøll PJ, Berube M, Allendorf FW (2007). Identification of management units using population genetic data. TREE.

[85] Waples RS, Gaggiotti O (2006). INVITED REVIEW: what is a population? an empirical evaluation of some genetic methods for identifying the number of gene pools and their degree of connectivity. Mol Ecol.

[86] Wilson GA, Rannala B (2003). Bayesian inference of recent migration rates using multilocus genotypes. Genetics.

[87] Faubet P, Waples RS, Gaggiotti OE (2007). Evaluating the performance of a multilocus Bayesian method for the estimation of migration rates. Mol Ecol.

[88] Wright S (1931). Evolution in mendelian populations. Genetics.

[89] Whitlock MC, McCauley DE (1999). Indirect measures of gene flow and migration: FST ≠ 1/(4 Nm + 1). Heredity.

[90] Beerli P, Bertorelle G, Bruford MW, Hauffe HC, Rizzoli A, Vernesi C (2001). How to use MIGRATE or why are Markov chain Monte Carlo programs difficult to use. Population genetics for animal conservation.

[91] Harvey MG, Brumfield RT (2015). Genomic variation in a widespread neotropical bird (xenops minutus) reveals divergence, population expansion, and gene flow. Mol Phylogenet Evol.

[92] Harvey MG, Smith BT, Glenn TC, Faircloth BC, Brumfield RT (2016). Sequence capture versus restriction site associated DNA sequencing for shallow systematics. Syst Biol.

[93] Heupink TH, van den Hoff J, Lambert DM (2012). King penguin population on Macquarie Island recovers ancient DNA diversity after heavy exploitation in historic times. Biol Lett.

[94] Hall KJ, Ehlers J, Gibbard PL (2004). Quaternary glaciations of the Sub-Antarctic Islands. Quaternary glaciations - extent and chronology part III.

[95] Gersonde R, Crosta X, Abelmann A, Armand L (2005). Sea-surface temperature and sea ice distribution of the Southern Ocean at the EPILOG last glacial maximum—a circum-Antarctic view based on siliceous microfossil records. Quat Sci Rev.

[96] Tavecchia G, Tenan S, Pradel R, Igual JM, Genovart M, Oro D. Climate-driven vital rates do not always mean climate-driven population. Glob Change Biol. 2016. doi:10.1111/gcb.13330 10.1111/gcb.13330 27279167

