# Supplementary figures and images for: Dispersal in the sub-Antarctic: king penguins show remarkably little population genetic differentiation across their range
Source: BMC Evol Biol. 2016 Oct 13;16:211. doi: 10.1186/s12862-016-0784-z (PMC5062852; doi:10.1186/s12862-016-0784-z)

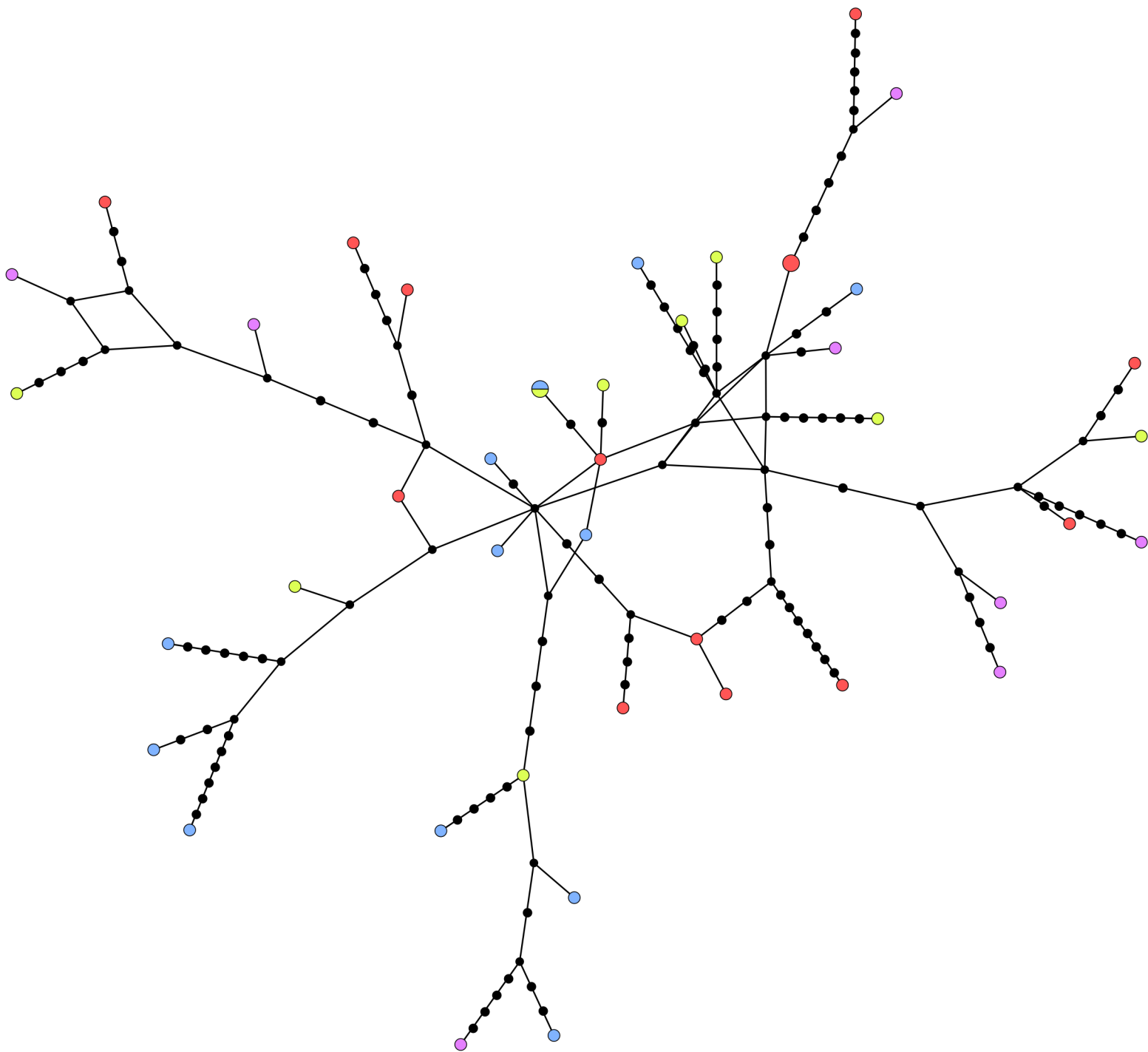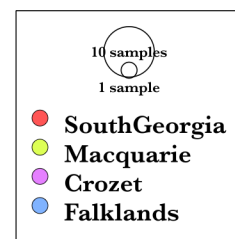

Supplement: Additional file 1: Figure S1. — Median-joining haplotype network of king penguin HVR sequences. (PDF 281 kb) [file 12862_2016_784_MOESM1_ESM.pdf]
